# Small vessel disease burden and intracerebral haemorrhage in patients taking oral anticoagulants

**DOI:** 10.1136/jnnp-2020-325299

**Published:** 2021-03-19

**Authors:** David J Seiffge, Duncan Wilson, Gareth Ambler, Gargi Banerjee, Isabel Charlotte Hostettler, Henry Houlden, Clare Shakeshaft, Hannah Cohen, Tarek A Yousry, Rustam Al-Shahi Salman, Gregory Lip, Martin M Brown, Keith Muir, H.R Jäger, David J Werring

**Affiliations:** 1 Department of Neurology and Stroke Center, Inselspital Universitatsspital Bern, Bern, BE, Switzerland; 2 Stroke Research Centre, UCL Queen Square Institute of Neurology, London, UK; 3 Department of Neurology and Stroke Center, University Hospital Basel, Basel, Switzerland; 4 New Zealand Brain Research Institute, University of Otago, Christchurch, New Zealand; 5 Department of Statistical Science, University College London, London, London, UK; 6 MRC Centre for Neuromuscular Diseases, UCL Institute of Neurology and National Hospital for Neurology and Neurosurgery, London, UK; 7 Haemostasis Research Unit, Department of Haematology, University College London, London, London, UK; 8 Neuroradiological Academic Unit, Department of Brain Repair & Rehabilitation, UCL Queen Square Institute of Neurology, London, UK; 9 Centre for Clinical Brain Sciences, University of Edinburgh Division of Medical and Radiological Sciences, Edinburgh, Edinburgh, UK; 10 Liverpool Centre for Cardiovascular Science, University of Liverpool and Liverpool Heart & Chest Hospital, Liverpool, Merseyside, UK; 11 Aalborg Aalborg Thrombosis Research UnitThrombosis Research Unit, Department of Clinical Medicine, Aalborg University, Aalborg, Denmark; 12 Institute of Neuroscience & Psychology, University of Glasgow and Queen Elizabeth University Hospital, Glasgow, UK

## Abstract

**Objective:**

We investigated the contribution of small vessel disease (SVD) to anticoagulant-associated intracerebral haemorrhage (ICH).

**Methods:**

Clinical Relevance of Microbleeds in Stroke-2 comprised two independent multicentre observation studies: first, a cross-sectional study of patients with ICH; and second, a prospective study of patients taking anticoagulants for atrial fibrillation (AF) after cerebral ischaemia. In patients with ICH, we compared SVD markers on CT and MRI according to prior anticoagulant therapy. In patients with AF and cerebral ischaemia treated with anticoagulants, we compared the rates of ICH and ischaemic stroke according to SVD burden score during 2 years follow-up.

**Results:**

We included 1030 patients with ICH (421 on anticoagulants), and 1447 patients with AF and cerebral ischaemia. Medium-to-high severity SVD was more prevalent in patients with anticoagulant-associated ICH (CT 56.1%, MRI 78.7%) than in those without prior anticoagulant therapy (CT 43.5%, p<0.001; MRI 64.5%, p=0.072). Leukoaraiosis and atrophy were more frequent and severe in ICH associated with prior anticoagulation. In the cerebral ischaemia cohort (779 with SVD), during 3366 patient-years of follow-up the rate of ICH was 0.56%/year (IQR 0.27–1.03) in patients with SVD, and 0.06%/year (IQR 0.00–0.35) in those without (p=0.001); ICH was independently associated with severity of SVD (HR 5.0, 95% CI 1.9 to 12.2, p=0.001), and was predicted by models including SVD (c-index 0.75, 95% CI 0.63 to 0.85).

**Conclusions:**

Medium-to-high severity SVD is associated with ICH occurring on anticoagulants, and independently predicts ICH in patients with AF taking anticoagulants; its absence identifies patients at low risk of ICH. Findings from these two complementary studies suggest that SVD is a contributory factor in ICH in patients taking anticoagulants and suggest that anticoagulation alone should no longer be regarded as a sufficient ‘cause’ of ICH.

**Trial registration:**

NCT02513316

## Introduction

Cerebral small vessel disease (SVD) accounts for the vast majority of non-traumatic spontaneous intracerebral haemorrhage (ICH), accounting for 77% in patients aged 70 years or younger in a study from Western Europe^1^ and 77%–85% of patients aged 18 years or older in a study from Western Europe and the USA.^2^ Meta-analyses^3^ of randomised controlled trials suggest that oral anticoagulants—vitamin K antagonists and direct oral anticoagulants (apixaban, dabigatran, edoxaban and rivaroxaban)—are a risk factor for ICH. However, there is no evidence that anticoagulants can cause arterial rupture, vascular fragility or inhibit vascular repair.^4 5^ In the context of many competing risk factors,^6 7^ the direct independent contribution of anticoagulation to ICH is unknown. Moreover, most ICHs on vitamin K antagonists occur within the therapeutic range^8^ and a recent study found that patients with ICH while taking rivaroxaban had low plasma levels.^9^ Despite these observations, oral anticoagulation is often regarded as a sufficient cause of ICH and has been used in clinical and mechanistic classification systems for ICH aetiology.^10–12^ Alternatively, it has been hypothesised that anticoagulation might exacerbate spontaneous ‘microbleeding’ due to SVD, resulting in symptomatic ICH.^13–15^

Several markers of SVD can be identified on neuroimaging.^16^ On MRI, cerebral microbleeds, cortical superficial siderosis, perivascular spaces, white matter hyperintensities, lacunes and atrophy can be diagnosed. On CT, leukoaraiosis, lacunes and atrophy can be measured. The overall burden of SVD can thus be assessed using scores based on MRI^17 18^ (presence of cerebral microbleeds, basal ganglia perivascular spaces, white matter hyperintensities and lacunes) or CT (presence of leukoaraiosis, lacunes and atrophy).

We hypothesised that cerebral SVD (causing brain small vessel fragility) is an important underlying contributory factor in ICH occurring in patients taking oral anticoagulants. Thus, SVD should: (1) be highly prevalent and associated with prior anticoagulant use in cross-sectional analyses of patients with ICH; and (2) predict ICH in prospective analyses of patients without ICH treated with oral anticoagulants. Moreover, if SVD is necessary for anticoagulant-associated ICH, then patients without any evidence of SVD prior to starting oral anticoagulation should have a very low rate of subsequent ICH.

With these considerations in mind, we analysed data from two independent cohorts to obtain complementary lines of evidence for a causal link between SVD and anticoagulant-related ICH through strength of association, consistency, biological gradient, temporality and coherence as described in the Bradford Hill criteria.^19^ We aimed to determine: (1) the association of SVD burden at baseline with future risk of ICH and ischaemic stroke in patients with atrial fibrillation (AF) exposed to oral anticoagulants for the first time; and (2) the prevalence and severity of SVD markers on CT and MRI and the overall SVD burden in patients with ICH on prior anticoagulant therapy compared with patients with ICH without prior anticoagulant therapy.

## Material and methods

### Study design and participants

The prospective observational ‘Clinical Relevance of Microbleeds in Stroke’ (CROMIS-2) Study enrolled adult patients (ie ≥18 years of age) at 79 centres in the UK and 1 centre in the Netherlands into two studies. In brief, in study 1 (‘atrial fibrillation cohort’) patients with presumed cardioembolic ischaemic stroke due to non-valvular AF started on oral anticoagulant therapy for secondary stroke prevention were enrolled into an inception cohort study with follow-up for ischaemic stroke and intracranial haemorrhage. We excluded patients if they could not undergo MRI at baseline, had a defined contraindication to oral anticoagulation therapy or had previously received therapeutic anticoagulation. In study 2 (‘intracerebral haemorrhage cohort’) we enrolled patients with imaging-confirmed non-traumatic ICH with or without treatment with oral anticoagulants (for any indication including AF) prior to onset. The trial protocol^20^ and the main analysis^13^ of study 1 (AF cohort) have been published previously. The current analysis follows a prespecified analysis plan (V.1.1 dated 31 October 2018 and approved by the Trial Steering Committee) using data from both substudies in a two-step analysis. Exclusion criteria included known underlying structural cause for ICH or major head trauma (causing loss of consciousness and thought to be sufficient to have caused the ICH) in the last 24 hours before presentation.

Patients with capacity gave informed written consent. When patients could not consent, we obtained written informed consent from a proxy as defined by relevant local legislation.

### Procedures

In study 1 (AF cohort) all patients underwent baseline brain MRI according to a predefined protocol parameter range,^20^ designed to detect relevant markers of cerebrovascular disease which required T2*-weighted gradient-recalled echo, axial T1-weighted, axial T2-weighted, coronal fluid-attenuated inversion recovery (FLAIR), and diffusion-weighted imaging with apparent diffusion coefficient maps. In study 2 (ICH cohort), all patients had intracranial haemorrhage confirmed on acute baseline non-contrast brain CT according to local standard clinical protocols; a subgroup of patients underwent additional brain MRI (‘intracerebral haemorrhage MRI subgroup’). We collected anonymised DICOM images of all patients at the core imaging analysis centre (Stroke Research Centre, UCL Queen Square Institute of Neurology). Imaging analysis of SVD markers on CT (ICH cohort) and MRI (AF cohort and ICH MRI subgroup) was performed by clinical research fellows (DW, GB and DJS) trained by a professor of neuroradiology (HRJ) according to an international consensus statement^16^ using validated scales and scores where available^21–27^ and as described in prior research.^13 28^ Raters were blinded to anticoagulation therapy status of the patients.

The following SVD markers were analysed: lacunes were identified and counted on axial non-contrast CT scans and on T2/FLAIR MRI sequences. Cerebral microbleeds were rated using blood-sensitive sequences and the validated Microbleed Anatomical Rating Scale.^25^ Cortical superficial siderosis was identified on blood-sensitive sequences and classified as either focal (involving ≤3 sulci) or disseminated (involving ≥4 sulci).^29^ White matter hyperintensities were rated on MRI T2/FLAIR in deep white matter and periventricular white matter sequences using the Fazekas Scale.^24 30^ Leukoaraiosis on CT was rated using the Van Swieten Scale in the anterior (plexus choroideus and cella media) and posterior (cella media and centrum semiovale) regions.^21^ Global cortical atrophy was rated using the Pasquier Scale on axial MRI T1/FLAIR images (T2 in the absence of FLAIR).^23^ Medial temporal lobe atrophy was rated using the Scheltens Visual Scale^26^ on MRI T1 or FLAIR coronal images. For both, the hemisphere contralateral to the intracerebral haemorrage was preferentially rated. Deep and cortical atrophy on CT was rated using a visual scale.^31 32^ MRI-visible perivascular spaces in the centrum semiovale and basal ganglia were defined and rated on T2/FLAIR sequences using a validated 4-point visual rating scale.^27 33^

The burden of SVD on MRI was rated using a validated SVD burden score with an ordinal scale from 0 to 4 by counting the presence of MRI features of SVD. One point is awarded for each of the following: presence of ≥1 lacunes and ≥1 cerebral microbleeds (1 point each if present), presence of perivascular spaces in the basal ganglia (grade 2–4 according to a previously published scale,^27^ 1 point if present) and presence of white matter hyperintensities (confluent deep white matter hyperintensities Fazekas Score 2 or 3 or irregular periventricular white matter hyperintensities extending into the deep white matter Fazekas Score 3; 1 point if present). The SVD burden score was categorised into SVD burden absent/below imaging detection level (score of 0) versus present (score of 1–4). Severity of the SVD burden was further categorised as none/low (score of 0), medium (score 1–2) and high (score of 3–4). Additionally, in the ICH cohort, the SVD burden was rated using a previously described CT-based burden score (using leukoaraiosis, lacunes and atrophy; score range 0–3)^32^ and similarly categorised into none/low burden (CT score of 0), medium (CT score of 1) and high (CT score of 2–3).

Baseline clinical data including demographics, risk factors, concomitant medication and characteristics of baseline ischaemic stroke (AF cohort) or ICH (ICH cohort) were collected by local study personnel using standardised forms as described in prior research.^13 34^ We calculated the CHA_2_DS_2_-VASc Score (**C** ongestive heart failure, **H** ypertension, **A** ge ≥ 75 y, **D** iabetes mellitus, **S** troke, **V** ascular disease, **A** ge 65-74 y and **S** ex **c** ategory, to predict ischaemic stroke)^35^ and the HAS-BLED^36^ Score (Hypertension, Abnormal renal/liver function, Age >= 65 years, Stroke, Bleeding, Labile INRs, Taking other drugs, Alcohol intake; to predict ICH) from clinical variables. In the AF cohort, follow-up information was obtained from patients and general practitioners at 6, 12 and 24 months via standardised structured postal questionnaires or telephone interviews.

### Outcomes

In the ICH cohort, the main variable of interest was prevalence and severity of SVD markers at ICH onset on CT (leukoaraiosis, lacunes and atrophy—summarised using the burden score). Secondary outcomes were prevalence and severity of SVD markers on MRI (cerebral microbleeds, cortical superficial siderosis, perivascular spaces, white matter hyerintensities, lacunes and atrophy) and the overall MRI SVD burden score (presence and severity as defined above).

In the AF cohort, the primary outcome was symptomatic ICH (defined as bleeding in the brain parenchyma, not including haemorrhage exclusively in subarachnoid or subdural spaces) confirmed on brain imaging during follow-up. Secondary outcome was ischaemic stroke confirmed on brain imaging during follow-up.

Primary and secondary outcome events in the AF cohort were adjudicated by a board of two professors of vascular neurology (DJW and MMB) and a clinical research fellow (DW). All adjudication was blinded to baseline MRI SVD burden. In cases of disagreement, we reached consensus after discussion.

### Statistics

The statistical analysis (DJS and GA) was conducted following a preplanned statistical analysis plan approved by the trial steering committee (V.1.1 dated 31 October 2018, not published). We conducted a two-stage analysis: in stage 1, (ICH cohort), we determined the presence and severity of SVD markers on CT and MRI and analysed their association with the type of ICH (with or without prior use of oral anticoagulants). Finally, we determined and compared the overall SVD burden using MRI among different types of ICH; in stage 2 (AF cohort), we compared the rate of ICH and ischaemic stroke during follow-up in patients with SVD burden on baseline MRI (MRI burden score of 1–4) and those free of SVD burden on baseline MRI (MRI burden score of 0). In the secondary analysis we compared the rate of ICH according to SVD burden severity (MRI burden score of 0=none/low burden; score of 1–2=medium burden; score of 3–4=igh burden).

In the ICH cohort, we divided patients into two groups: (1) Patients with intake of oral anticoagulants prior to onset of ICH (anticoagulant-associated ICH); and (2) Patients without prior intake oral anticoagulants. We compared demographic and clinical baseline characteristics among groups within both cohorts using Fisher’s/Pearson χ^2^ test for categorical variables and the t-test/Mann-Whitney U test for continuous variables as appropriate. We described prevalence and severity of SVD markers on CT and MRI using descriptive statistics, Pearson χ^2^ test and appropriate graphs. We compared prevalence, distribution and mean value of the SVD burden score using descriptive statistics, Fisher’s/Pearson χ^2^ test and graphs as appropriate. We assessed the association of prior oral anticoagulation therapy (vs non-prior anticoagulation therapy) and MRI-SVD burden score using univariable and multivariable logistic regression models adjusting for predefined variables. We assessed the association between prior anticoagulation (vs no prior anticoagulation) and SVD markers on CT and MRI using univariable and multivariate analysis adjusting for predefined variables. For SVD markers on CT, we adjusted our between-group comparisons for the following predefined variables because of their association with cerebrovascular disease including SVD: age, history of ischaemic stroke, history of ICH, AF, myocardial infarction, diabetes mellitus, hypertension, hypercholesterolaemia, current smoking and concomitant medication with antiplatelets agents, antihypertensive agents and statins. Due to a smaller sample size, we used only the following predefined variables for multivariate analysis of the SVD markers on MRI and the MRI SVD burden score: age, hypertension, diabetes mellitus, AF, hypercholesterolaemia, current smoking and antihypertensive treatment. We present ORs with 95% CIs for all models.

In the AF cohort, we divided patients into groups according to SVD burden severity: none/low SVD burden (MRI burden score of 0), medium SVD burden (MRI burden score of 1–2) or high SVD burden (MRI burden score of 3–4). We compared baseline demographics and risk factor profiles between the aforementioned SVD burden groups. We calculated annualised event rates for ICH and ischaemic stroke in the different groups (observed events/patient-years of follow-up). We estimated Kaplan-Meier curves according to SVD burden groups. The association between severity of SVD burden with ICH and ischaemic stroke during follow-up was analysed using Cox proportional hazards models. We constructed univariable and two multivariable models to predict ICH: multivariable model 1 included the HAS-BLED^36^ Score (a score estimating the 1-year risk of major bleeding in patients with AF using clinical risk factors and demographics) which was dichotomised in two categories: low risk (score 0–2) and high risk (score ≥3). For ischaemic stroke as outcome, we used the CHA_2_DS_2_-VASc^35^ Score (a score estimating the risk of systemic embolism) dichotomised at the median (5) of our cohort into medium (score of 2–4) and high (score of ≥5) risk. Multivariable model 2 included age and history of hypertension. We calculated HRs and 95% CI. We investigated the predictive ability of SVD burden using a Cox model and performed internal validation using bootstrapping and assessed the calibration and discrimination of this model. All statistical analysis was conducted using STATA V.15.0. This study is registered with ClinicalTrials.gov, number NCT02513316.

### Role of the funding source

Neither the funders nor the sponsor had input into study design; data collection, data analysis, data interpretation; writing of the report; or the decision to submit the paper for publication. The corresponding author had full access to all the data in the study and had final responsibility for the decision to submit for publication.

### Data availability

Anonymised data are available on request from the corresponding author for any reasonable request and after clearance by the competent ethics committee.

## Results

**Figure 1:**
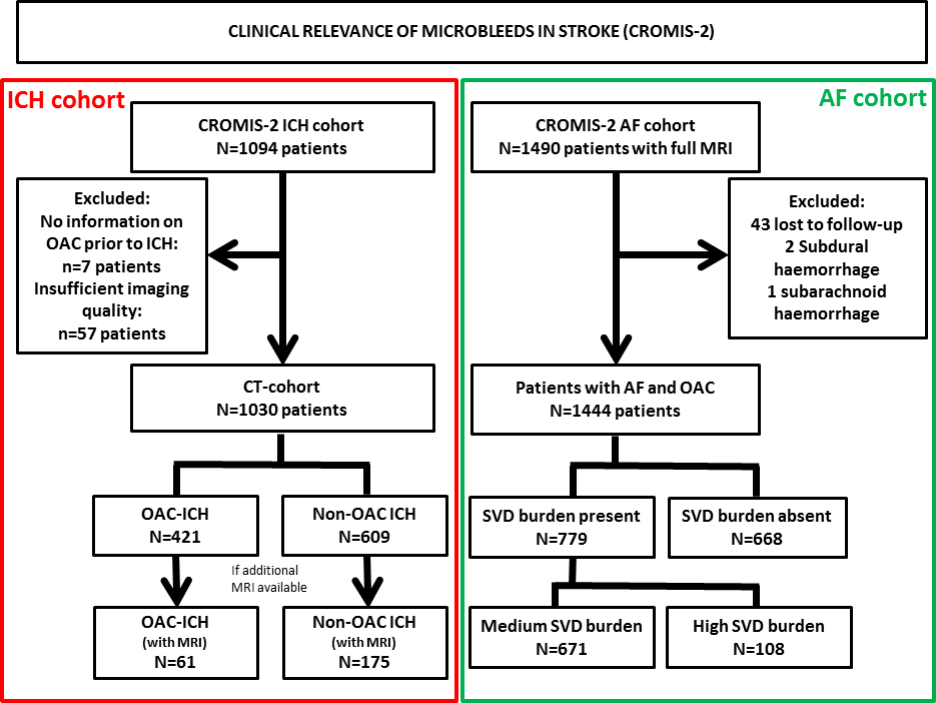
Study flow chart. AF, atrialfibrillation; CROMIS-2, Clinical Relevance of Microbleedsin Stroke; ICH, intracerebral haemorrhage; SVD, small vessel disease; OAC, oral anticoagulation.

We included 1030 patients in the ICH cohort and 1447 patients in the AF cohort. Figure 1 displays the study flow chart.

### ICH cohort—baseline characteristics

In the ICH cohort, among 1030 patients, 421 patients had ICH with prior oral anticoagulant therapy (40.9%) and 609 patients had ICH without prior anticoagulant therapy (59.1%). The median age of the ICH cohort was 76 years (IQR 66–83 years), the median haematoma volume 7 mL (IQR 2–18 mL) and 439 patients (42.6%) were female. Among the 421 patients on prior anticoagulation, 28 patients (6.7%) were taking direct oral anticoagulants (dabigatran, rivaroxaban or apixaban). All other patients were taking vitamin K antagonists. Among the entire cohort, 236 patients (on prior anticoagulant therapy: 61 patients and without prior anticoagulant therapy: 175 patients) had additional MRI available (MRI substudy cohort).

Table 1 displays the baseline characteristics of patients in the ICH cohort. Patients on prior anticoagulation therapy were significantly older and had more cerebrovascular risk factors (ie, history of ischaemic stroke, AF, hypertension, hypercholesterinaemia and diabetes mellitus) while patients without prior anticoagulation therapy were more often current smokers. Baseline haemorrhage volume, National institute of health stroke score (NIHSS) and Glasgow coma scale (GCS) were similar in both groups. Haematoma location was less often deep (prior anticoagulation: 188 patients/44.7% vs no prior anticoagulation: 337 patients/55.3%) and more often lobar (prior anticoagulation: 175 patients/41.6% vs no prior anticoagulation 230 patients/33.8%) and cerebellar (prior anticoagulation: 35 patients/8.3% vs no prior anticoagulation 23 patients/3.8%, p<0.001 for haemorrhage location) in patients on prior anticoagulation. Intraventricular haemorrhage was more often in patients with prior anticoagulation therapy (112 patients, 26.6%) compared with without prior anticoagulation therapy (100 patients, 16.4%, p<0.001). Overall, patients with deep haemorrhage had more often intraventricular expansion (n=141, 26.9%) compared to patients with lobar haemorrhage (n=54, 13.3%).

**Table 1:** Baseline characteristics of the intracerebral haemorrhage cohort (all patients with intracerebral haemorrhage, comparing patients taking oral anticoagulants prior to ICH onset with those patients not taking anticoagulants) and atrial fibrillation cohort (all patients with ischaemic stroke, atrial fibrillation and oral anticoagulant therapy comparing patients with none–low small vessel disease burden (small vessel disease absent) to those with medium–high small vessel disease burden (small vessel disease present))

		Intracerebral haemorrhage cohort			Atrial fibrillation cohort		
		On prior anticoagulation therapy (n=421)	Without prior anticoagulation therapy (n=609)	P value	Small vessel disease burden present (n=779)	Small vessel disease burden absent (n=668)	P value
Age		79 (72–84)	72 (62–80)	<0.001	80 (73–85)	73 (66–80)	<0.001
Female sex		175 (41.6%)	264 (43.4%)	0.548	336 (43.1%)	275 (41.2%)	0.451
NIHSS on admission		6 (3–13)	8 (4–13)	0.115	4 (2–9)	5 (2–11)	0.169
GCS on admission		15 (14–15)	15 (14–15)	0.126	n/a	n/a	n/a
Haemorrhage volume in ml	All locations	7.2 (2.4–20.6)	6.9 (2.1–16.0)	0.198	n/a	n/a	n/a
	Deep location	5.5 (1.6–12.2)	4.4 (1.6–10.4)	0.237			
	Lobar location	17.5 (5.0–35.3)	15.0 (6.7–31.7)	0.760			
INR on admission		2.3 (1.8–3.0)	1.0 (1.0–1.1)	<0.001	n/a	n/a	n/a
***Medical history***							
History of intracerebral haemorrhage		11 (2.6%)	32 (5.3%)	0.028	5 (0.7%)	3 (0.5%)	0.624
History of ischaemic stroke		75 (17.8%)	58 (9.5%)	<0.001	99 (12.9%)	41 (6.3%)	<0.001
Atrial fibrillation		326 (79.1%)	33 (6.1%)	<0.001	779 (100%)	668 (100%)	n/a
Hypertension		310 (73.6%)	372 (61.1%)	<0.001	541 (71.1%)	365 (55.0%)	<0.001
Myocardial infarction		51 (12.1%)	35 (5.8%)	<0.001	87 (11.3%)	44 (6.6%)	0.002
Hypercholesterolaemia		212 (50.4%)	237 (38.9%)	<0.001	344 (44.9%)	293 (44.4%)	0.853
Current smoker		24 (5.7%)	84 (13.8%)	<0.001	79 (10.5%)	80 (12.2%)	0.587
Diabetes mellitus		99 (23.5%)	90 (14.8%)	<0.001	135 (17.3%)	107 (16.1%)	0.521
Antiplatelet agent therapy		51 (12.1%)	208 (34.2%)	<0.001	33 (5.2%)	43 (5.6%)	0.568

### Prevalence and severity of SVD markers among patients with intracerebral haemorrhage and prior oral anticoagulation therapy

**Figure 2:**
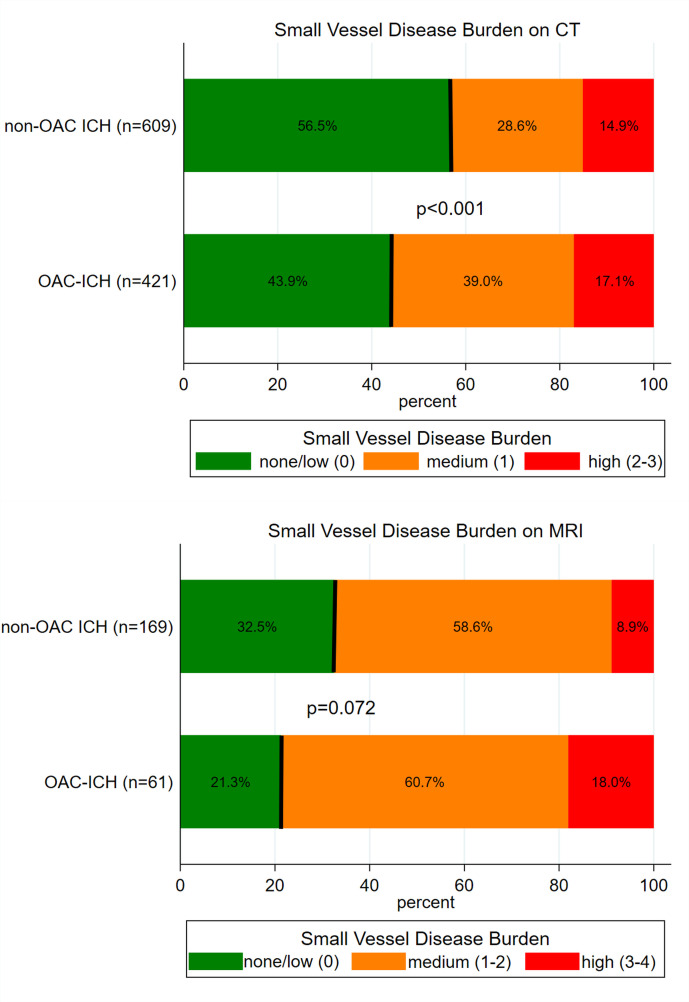
Small vessel disease burden on MRI in patients with intracerebral haemorrhage (ICH) with prior anticoagulation therapy compared with those without prior anticoagulation therapy.

The prevalence of medium-to-high severity SVD on CT and MRI was higher in those taking prior anticoagulants: on CT, a medium-to-high burden of SVD (measured using the CT burden score^32^ was present in 236 of 421 patients with prior anticoagulant therapy (56.1%) and 265 of 609 patients without prior anticoagulant therapy (43.9%, p<0.001, figure 2)). On MRI, a medium-to-high burden of SVD (measured using the MRI burden score^18^ was present in 48 of 61 patients with prior anticoagulant therapy (78.7%) and 114 of 169 patients without prior anticoagulant therapy (64.5%, p=0.072, figure 2)). In univariable analyses, prior anticoagulant therapy was significantly associated with higher SVD burden (unadjusted OR 1.5, 95% CI 1.1 to 1.9, p=0.007), but not after adjusting for confounders (adjusted OR 1.2, 95% CI 0.7 to 2.0, p=0.436). A post hoc sensitivity analysis among patients with deep haemorrhage confirmed this finding (adjusted OR: 0.99; 95% CI 0.51 to 1.91). Among patients on vitamin-K antagonist treatment with available international normalized ratio (INR) at haemorrhage onset (n=388 of 393 patients, 98.7%), the percentage of patients with medium-to-high SVD burden on CT was 60.6% among patients with INR <2.0, 48.2% among patients with INR 2.0–3.0 and 49.2% among patients with INR >3.0 (p=0.2).

Leukoaraiosis (on CT) and white matter lesions (on MRI), cortical atrophy on CT and MRI, and perivascular spaces in the basal ganglia were more prevalent and severe in patients with prior anticoagulant therapy (table 2). There was no difference in lacunes (on either CT or MRI), perivascular spaces in the centrum semiovale, cerebral microbleeds, siderosis or medial temporal lobe atrophy (table 2). However, after adjusting for potential confounders, there were no statistically significant differences in any SVD marker on CT or MRI between patients with prior anticoagulant therapy and those without (table 3).

**Table 2:** Distribution of different markers of small vessel disease on CT and MRI in patients with intracerebral haemorrhage comparing patients with prior anticoagulation therapy and those without

CT cohort				
		*Prior anticoagulation therapy (n=421*)	*No prior anticoagulation therapy (n=609*)	*P value*
***Leukoaraiosis (van Swieten Score)***				
Anterior region	0	221 (52.5%)	365 (59.9%)	0.059
	1	120 (28.5%)	149 (24.5%)	
	2	80 (19.0%)	95 (15.6%)	
Posterior region	0	223 (53.0%)	373 (61.2%)	0.023
	1	77 (18.3%)	84 (13.8%)	
	2	121 (28.7%)	152 (25.0%)	
***Lacunes***				
Number of lacunes	None	385 (91.5%)	545 (89.5%)	0.373
	1 lacuna	25 (5.9%)	50 (8.2%)	
	≥1 lacunes	11 (2.6%)	14 (2.3%)	
***Atrophy***				
Cortical atrophy	Absent	25 (5.9%)	137 (22.5%)	<0.001
	Moderate	299 (71.0%)	371 (60.9%)	
	Severe	97 (23.0%)	101 (16.6%)	
Deep atrophy	Absent	76 (18.1%)	190 (31.2%)	<0.001
	Moderate	228 (54.2%)	302 (49.6%)	
	Severe	117 (27.8%)	117 (19.2%)	
**MRI cohort**				
		***Prior anticoagulation therapy (n=61)***	***No prior anticoagulation therapy (n=175)***	
***White matter hyperintensities (Fazekas Scale)***				
Periventricular white matter	0	1 (1.6%)	12 (7.1%)	0.065
	1	19 (31.2%)	73 (43.2%)	
	2	28 (45.9%)	63 (37.3%)	
	3	12 (21.3%)	21 (12.4%)	
Deep white matter	0	19 (31.2)	85 (50.3%)	0.008
	1	16 (26.3%)	38 (22.5%)	
	2	14 (23.0%)	35 (20.7%)	
	3	12 (19.7%)	11 (6.5%)	
***Lacunes***				
Number of lacunes	No	46 (79.3%)	124 (77.0%)	0.862
	1	8 (13.8%)	27 (16.8%)	
	≥2	4 (6.9%)	10 (6.2%)	
***Atrophy***				
Global cortical atrophy	Absent	19 (31.2%)	99 (57.9%)	0.001
	Moderate	30 (49.2%)	48 (28.1%)	
	Severe	12 (19.7%)	24 (14.0%)	
Medial temporal atrophy	Absent	10 (21.7%)	38 (27.3%)	0.429
	Moderate	20 (43.5%)	66 (47.5%)	
	Severe	16 (34.8%)	35 (25.2%)	
***Cerebral microbleeds***				
Number	None	26 (42.6%)	94 (53.7%)	0.326
	1	7 (11.5%)	17 (9.7%)	
	≥2	28 (45.9%)	64 (36.6%)	
***Cortical superficial siderosis***				
None		45 (88.2%)	134 (90.5%)	0.889
Focal		4 (7.8%)	9 (6.1%)	
Disseminated		2 (3.9%)	5 (3.4%)	
***Perivascular spaces***				
Centrum semiovale	None	5 (8.2%)	14 (8.2%)	0.928
	Moderate	31 (50.8%)	91 (53.5%)	
	Severe	25 (41.0%)	65 (38.2%)	
Basal ganglia	None	7 (11.5%)	27 (15.9%)	0.001
	Moderate	37 (60.7%)	128 (75.3%)	
	Severe	17 (27.9%)	15 (8.8%)	

**Table 3:** Unadjusted and adjusted odds ratios for small vessel disease markers on CT and MRI in patients with intracerebral haemorrhage and prior anticoagulation therapy (vs no prior anticoagulation therapy)

			Unadjusted OR	P value	Adjusted* OR	P value
***Leukoaraiosis/white matter lesions***						
CT	Anterior region		1.2 (1.0–1.4)	0.023	0.8 (0.6–1.1)	0.209
	Posterior region		1.2 (1.0–1.3)	0.038	0.8 (0.6–1.1)	0.234
MRI	Periventricular white matter		1.7 (1.2–2.4)	0.003	1.2 (0.6–2.3)	0.656
	Deep white matter		1.6 (1.2–2.1)	0.001	1.3 (0.8–2.2)	0.264
***Lacunes***						
CT	Any lacunes		0.8 (0.5–1.2)	0.274	1.0 (0.5–2.2)	0.948
MRI	Any lacunes		1.5 (0.5–4.7)	0.451	0.6 (0.1–7.8)	0.666
***Atrophy***						
CT	Cortical atrophy		2.0 (1.6–2.5)	<0.001	0.9 (0.6–1.4)	0.671
	Deep atrophy		1.6 (1.3–1.9)	<0.001	1.0 (0.7–1.5)	0.837
MRI	Global cortical atrophy		1.7 (1.2–2.3)	0.004	0.6 (0.3–1.4)	0.267
	Medial temporal lobe		1.2 (0.9–1.7)	0.200	0.7 (0.4–1.2)	0.187
***Cerebral microbleeds, cortical superficial siderosis and perivascular spaces***						
MRI	Perivascular spaces	Centrum semiovale	0.9 (0.7–1.2)	0.567	1.2 (0.7–2.0)	0.589
		Basal ganglia	2.0 (1.2–3.2)	0.006	1.5 (0.6–3.6)	0.393
	Any cortical superficial siderosis		1.3 (0.4–4.2)	0.710	1.1 (0.1–1.7)	0.118
	Any cerebral microbleeds		1.6 (0.9–2.8)	0.098	1.8 (0.6–5.0)	0.279

*All CT analysis were adjusted for age, history of ischaemic stroke, history of ICH, atrial fibrillation, myocardial infarction, diabetes mellitus, hypertension, hypercholesterolaemia, current smoking and concomitant medication with antiplatelet agents, antihypertensive agents and statins.

*All MRI analysis were adjusted for age, hypertension, diabetes mellitus, atrial fibrillation, hypercholesterolaemia, current smoking and antihypertensive treatment.

### AF cohort—baseline characteristics

Among 1447 patients in the AF cohort, SVD was absent (burden score of 0) in 668 patients (46.2%) and present (burden score of 1–4) in 779 patients (53.8%). Among those with SVD present, 671 patients (86.1%) had medium burden (burden score 1–2) and 108 patients (13.9%) had high burden (burden score of 3–4). The median age of the AF cohort was 77 years (IQR 69–83 years) and 611 patients (42.2 %) were female. There was no difference regarding treatment with direct oral anticoagulants between both groups (SVD burden present: 281 (37.6%), SVD burden absent: 244 (37.4%), p=0.840).

Table 1 displays baseline characteristics of patients with SVD present compared with those with no markers of SVD on MRI (measured using the SVD burden score). Patients with SVD were older, had higher HAS-BLED (median HAS-BLED Score: SVD burden present 4, IQR 3–4 and SVD burden absent 3, IQR 3–4, p=0.004) and CHA_2_DS_2_-VASc (median CHA_2_DS_2_-VASc Score: SVD burden present 5, IQR 4–6 and SVD burden absent 5, IQR 3–5, p<0.001) Scores and more often a history of ischaemic stroke, hypertension and myocardial infarction. Overall, 1210 of 1447 patients (83.6%) in the AF cohort had a HAS-BLED Score of ≥3 (indicating high risk of bleeding).

During the follow-up of 3366 patient-years, there were 11 ICHs and 56 ischaemic strokes. The annual rate of ICH was 0.33%/year (95% CI 0.16% to 0.58%); the annualised rate of ischaemic stroke was 1.66%/year (95% CI 1.26% to 2.15%).

### SVD burden and future risk of ICH

**Figure 3:**
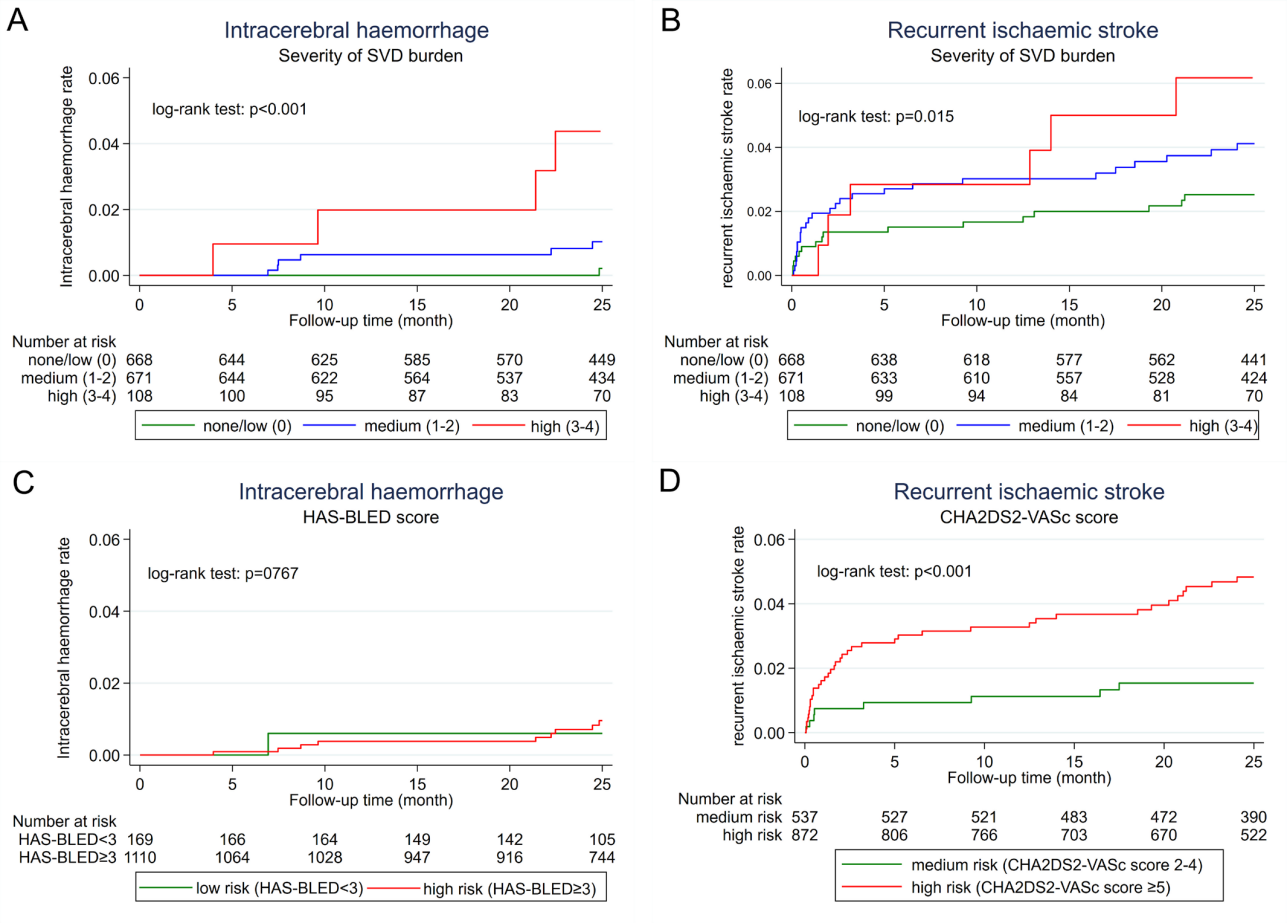
Kaplan-Meier curves of intracerebral haemorrhage (A, C) and recurrent ischaemic (B, D) stroke according to small vessel disease (SVD) burden (A, B), the HAS-BLED score (for intracerebral haemorrhage) (C) and the CHA2DS2-VASc (for recurrent ischaemic stroke) (D).

ICH was more frequent in patients with SVD (p<0.001) using the log-rank test for equality of survivor functions (figure 3A) and the annualised rate increased with SVD severity (table 4). The severity of SVD burden was associated with ICH in univariable regression analysis (HR 4.8, 95% CI 1.9 to 12.0, p=0.001) which was maintained in multivariable analysis adjusting for HAS-BLED Score (HR 5.0, 95% CI 1.9 to 12.2, p=0.001) or for age and hypertension (HR 4.6, 95% CI 1.8 to 11.8, p=0.001).

**Table 4:** Total events, annualised event rate and univariable and multivariable association of presence/absence and severity of small vessel disease (SVD) burden and intracerebral haemorrhage and recurrent ischaemic stroke

		*Follow-up time*	*Events*	*Annual event rate in %/year*	*Univariable*		Multivariable 1*		*Multivariable 2*	
					HR	P value	HR	P value	HR	P value
***Intracerebral haemorrhage***										
Severity of SVD	None/low (0)	1586	1	0.06 (0.00–0.35)	1 (ref)		1 (ref)			
	Medium (1–2)	1535	6	0.39 (0.14–0.85)	4.8 (1.9–12.0)	0.001	5.0 (1.9–12.2)	0.001	4.6 (1.8–11.8)	0.001
	High (3–4)	245	4	1.63 (0.45–4.13)						
***Recurrent ischaemic stroke***										
Severity of SVD	None/low (0)	1586	17	1.07 (0.63–1.71)	1 (ref)		1 (ref)		1 (ref)	
	Medium (1–2)	1535	32	2.08 (1.43–2.93)	1.7 (1.1–2.5)	0.010	1.5 (1.0–2.3)	0.049	1.5 (1.0–2.2)	0.075
	High (3–4)	245	7	2.86 (1.16–5.80)						

*Multivariate model 1: HAS-BLED Score dichotomised in two categories: low risk (score 0–2) and high risk (score ≥3) for intracerebral haemorrhage and for CHA_2_DS_2_-VASc Score dichotomised in two categories: medium risk (score 2–4) and high risk (≥5) for recurrent ischaemic stroke.

Multivariate model 2: age and history of hypertension.

By contrast, using the HAS-BLED Score (data available in 1279 patients, 87.7%, figure 3B), there was no statistically significant difference between patients with a HAS-BLED Score of ≥3 indicating high risk of bleeding (n=1110 patients, 2589 patient-years of follow-up, 9 ICH during follow-up, annualised rate 0.35%/year, 95% CI 0.16 to 0.66) compared with patients with a HAS-BLED Score of <3 indicating a low risk of bleeding (n=169, 386 patient-years of follow-up, 1 ICH during follow-up, annualised rate 0.26%/year, 95% CI 0.01 to 1.44).

Recurrent ischaemic stroke was more frequent in patients with SVD (p=0.015) using the log-rank test (figure 3C). The severity of SVD burden was associated with recurrent ischaemic stroke in univariable (p=0.01, table 4) analysis. In multivariable analysis adjusting for the CHA_2_DS_2_-VASc Score (p=0.049) or age and hypertension (p=0.075), there was weak evidence for an increased risk of recurrent ischaemic stroke. Using the CHA_2_DS_2_-VASc Score (data available in 1409 patients=95.4%, figure 3D), the risk of recurrent ischaemic stroke was more frequent in patients with a score of ≥5 indicating high risk (n=872, 1899 patient-years of follow-up, 47 recurrent ischaemic strokes, annualised rate 2.42%/year, 95% CI 1.80 to 3.18) compared with those with a score of 2–4 indicating medium risk (n=537, 8 recurrent ischaemic strokes, 1334 patient-years of follow-up, annualised rate 0.60%/year, 95% CI 0.28 to 1.14).

After adjusting for optimism, the c-index for the unadjusted model for ICH using severity of SVD burden (c-index: 0.75, 95% CI 0.63 to 0.85) was higher than a model using the HAS-BLED Score (c-index: 0.51, 95% CI 0.39 to 0.57). Multivariable models including severity of SVD burden and the HAS-BLED Score (model 1; c-index: 0.73, 95% CI 0.64 to 0.86) or age and hypertension (model 2; c-index: 0.71, 95% CI 0.63 to 0.84) did not further improve prediction of ICH (all p>0.05). The unadjusted model exhibited the best calibration (calibration slope near to 1).

## Discussion

We show that: (1) imaging markers of SVD are highly prevalent and severe in patients with ICH both with and without prior anticoagulant therapy, but are more common in those with ICH associated with anticoagulation; (2) the severity of SVD (measured with a four-item MRI score) independently predicts ICH in patients with AF taking anticoagulants; (3) the absence of SVD identifies patients at very low risk of ICH on anticoagulant therapy; and (4) prediction models for ICH including severity of SVD burden have good discrimination, but a commonly used clinical score (HAS-BLED) performs poorly.

Our findings confirm and expand current knowledge on the role of SVD in ICH in patients taking anticoagulants, and strongly suggest SVD as an important contributory cause of ICH in these patients. This study is the first to compare the burden of SVD markers among patients with ICH with and without prior anticoagulation therapy. The majority of patients who had ICH with prior anticoagulant therapy had SVD burden detectable on CT (56.1%) or MRI (78.7%). Moreover, medium-to-high severity SVD was more prevalent in patients with anticoagulant-associated ICH (CT 56.1%, MRI 78.7%) than in those without prior anticoagulant therapy (CT 43.5%, p<0.001; MRI 64.5%, p=0.072). Since SVD is the commonest cause of spontaneous ICH overall, our findings are consistent with the hypothesis that SVD is contributory or causal for ICH occurring during anticoagulant therapy. We can only speculate about the possible aetiology in those patients without SVD burden. However, on MRI, there were subtle signs of SVD present in all but two patients which did not reach the level to score one point on the respective burden score scale (eg, Fazekas grade I), so SVD is likely to be contributory or causal in these patients, too. A recent meta-analysis found haematoma location—as a surrogate marker for underlying type of SVD (cerebral amyloid angiopathy or hypertensive deep perforator arteriolopathy)—to be more frequently cerebellar and intraventricular in ICH associated with the use of anticoagulants^37^ possibly reflecting differences in the spectrum of underlying type of SVD. We systematically assessed prevalence, severity and burden of SVD lesions, but future studies are needed to determine whether the spectrum of SVD (ie, deep perforator arteriolopathy vs cerebral amyloid angiopathy) differs between patients with ICH and prior anticoagulant therapy and those without.

Recent data from CROMIS-2^13 38^ found that cerebral microbleeds—a key haemorrhagic imaging marker of SVD—are associated with a risk of ICH in patients taking anticoagulant therapy. However, cerebral microbleeds alone could not explain all ICHs occurring during follow-up. Our current analysis builds on these previous findings by incorporating a full range of ischaemic and haemorrhagic SVD imaging markers (which are considered to share similar pathophysiological mechanisms^16 39^) combined in an SVD burden score. In our AF cohort, within 2 years of follow-up, ICH occurred almost exclusively in patients with medium or high SVD burden present on baseline MRI, highlighting the importance of SVD as a risk marker (and furthermore, almost a necessary factor) for ICH. Only one patient had ICH in the group classified as having none or low SVD burden on baseline MRI, though this patient did have white matter lesions and enlarged perivascular spaces not fulfilling the criteria to score one point on the SVD burden score. In the absence of follow-up MRI in this patient, it is possible that SVD progressed during the 24-month observation period. We observed an increased ischaemic stroke risk with increasing SVD burden, suggesting that this may contribute to ischaemic stroke recurrence.

A major strength of our approach when compared with clinical risk factor-based scores, such as HAS-BLED, is that we directly visualised the SVD lesions that are hypothesised to cause most spontaneous ICH. In our AF cohort, according to clinical risk factors and the HAS-BLED Score, 84% of the patients were at high risk (a score of 3 or higher). Using the severity of SVD burden provided higher precision predicting ICH compared with clinical risk factors.

Our study has the following strengths: we undertook two prospective multicentre studies with different designs which provide complementary and generalisable evidence for a causal link between SVD and ICH in patients taking oral anticoagulants (through strength of association, consistency, biological gradient, and temporality and coherence as described in the Bradford Hill criteria).^19^ Both studies comprised a large number of well-phenotyped patients enrolled in 79 centres across the UK (and one in the Netherlands).

Our study has potential limitations: (1) despite our detailed clinical and neuroimaging phenotyping, unmeasured factors (eg, genetic variants) might have influenced our results; (2) due to a relatively low rate of ICH in the AF cohort (n=11), we had limited statistical precision and power, so were unable to correct multivariable analysis for more than two co-variables; (3) both cohorts are likely to be affected by selection bias as participation in both studies required written informed consent, making it less likely that patients with very severe neurological deficits were enrolled (moreover, in the AF cohort, all patients had to have MRI and it is therefore likely that the index stroke was less severe); (4) in both studies, the vast majority of patients taking anticoagulants received vitamin K antagonist therapy and not all results may apply to patients taking direct oral anticoagulants; (5) the ICH cohort was specifically designed to investigate the characteristics of patients with ICH on anticoagulant therapy and enrolment of these patients was favoured to enrich the cohort, leading to a selection bias towards these patients; (6) among patients in the ICH cohort, those with MRI were younger and with less neurological severity; this probably led to an underestimation of the SVD burden; and (7) technical aspects including field strength and sequence type (SWI vs GRE) were heterogenous throughout study sites which is a source of bias regarding detection of cerebral microbleeds.^40^

Our study provides new evidence that SVD is an important cause of ICH among patients taking anticoagulants, and further advocates against the erroneous classification of anticoagulant use as a sufficient ‘cause’ of ICH. Future studies are needed to determine whether ICH in patients taking prior anticoagulant therapy is more closely associated with the main known types of sporadic cerebral SVD—hypertensive deep perforator arteriolopathy or cerebral amyloid angiopathy. Importantly, in patients with AF taking oral anticoagulant therapy, MRI might help to estimate the risk of ICH: the absence of SVD on MRI can potentially reassure patients, carers and treating physicians of a very low risk of ICH. However, our data do not support withholding anticoagulant therapy in patients with AF and SVD burden on MRI as these patients are also at high risk of ischaemic stroke. Future research is needed to determine whether alternative treatments (eg, left atrial appendage closure) might be a safe and effective option in patients with AF and high SVD burden.

## Data Availability

Anonymised data are available upon reasonable request from the corresponding author and after clearance by the competent ethics committee.
